# Fetomaternal Outcome Following Previous Spontaneous Abortion: An Observational Study

**DOI:** 10.7759/cureus.111864

**Published:** 2026-07-01

**Authors:** Ponmani Priya Veeramani Venkatachalapathi, Uthpala Vadakaluru, Arivarasan Barathi

**Affiliations:** 1 Obstetrics and Gynecology, Mahatma Gandhi Medical College and Research Institute, Puducherry, IND; 2 Community Medicine, ESIC Medical College and Hospital, Chennai, IND

**Keywords:** fetal maternal outcomes, gdm- gestational diabetes mellitus, gestational hypertension, neonatal intensive care unit (nicu) admission, pregnancy outcome . prevelance

## Abstract

Background: Spontaneous abortion (SAB, miscarriage) affects and may influence outcomes in subsequent pregnancies. This study aimed to assess maternal and fetal outcomes in women with a history of SAB who conceived a subsequent pregnancy.

Methodology: A hospital‑based observational study was conducted at Mahatma Gandhi Medical College and Research Institute, Puducherry, over 18 months. Ninety‑six women aged 18-35 years with at least one previous SAB were enrolled. Maternal outcomes (gestational hypertension, diabetes mellitus, placental complications, and mode of delivery) and fetal/neonatal outcomes (fetal growth retardation, birth weight, Apgar score, and neonatal intensive care unit (NICU) admission) were recorded. Comparisons were made between single (82, 85.4%) and recurrent (≥2 abortions; 14, 14.6%) subgroups.

Results: A total of 96 pregnant women with a history of SAB were included. The mean maternal age was 27.74 ± 4.81 years, and most participants were aged <30 years (65, 67.7%) and had conceived spontaneously (88, 91.7%). Recurrent SAB was present in 14 (14.6%) women. Gestational diabetes mellitus and gestational hypertension were observed in 17 (17.7%) and 12 (12.5%) women, respectively. Preterm delivery occurred in 12 (12.5%) pregnancies, while 58 (60.4%) women underwent cesarean section. Fetal growth restriction was identified in 12 (12.5%) cases, and NICU admission was required in 18 (18.8%) neonates. Low birth weight, low APGAR score, and meconium aspiration syndrome were each observed in 1 (1.0%) neonate, with no intrauterine deaths recorded. Recurrent abortion was associated with higher rates of preterm birth (21.4% vs. 11.0%; *P *= 0.375) and NICU admission (28.6% vs. 17.1%; *P *= 0.291), although these associations were not statistically significant. Preterm delivery was significantly associated with NICU admission (41.7% vs. 15.5%; *P *= 0.043).

Conclusions: Pregnancy after SAB generally results in favorable outcomes with no intrauterine deaths and high rates of normal birth weight and Apgar scores. However, clinically significant risks of gestational diabetes, hypertension, fetal growth retardation, high cesarean section rates, and NICU admissions persist. Structured antenatal surveillance and individualized care are recommended for this population.

## Introduction

Loss of pregnancy, especially when unplanned and spontaneous, remains a major concern in obstetric care because it can have both short‑ and long‑term consequences for the mother. Spontaneous abortion (SAB) (miscarriage) affects approximately 10%-20% of clinically recognized pregnancies and carries wide‑ranging psychological, physical, and reproductive implications. One of the central questions is how a miscarriage influences subsequent pregnancies. Several studies have reported that women who suffer an SAB have an increased risk of complications in later pregnancies, including preterm delivery, intrauterine growth restriction (IUGR), and low birth weight [1].

Within the field of reproductive medicine, an increasing number of publications stress the importance of previous reproductive loss on future gestations. The risk of a second miscarriage or medically indicated termination may be significantly increased after a history of second‑trimester loss, and adverse maternal and neonatal outcomes around delivery may be associated with such a history [2]. This is particularly relevant for women with recurrent pregnancy loss (RPL, defined as two or more losses), which may indicate underlying anatomical, genetic, endocrine, or immunological abnormalities [3].

Recent population‑based studies have shed light on how previous spontaneous and induced abortions affect perinatal outcome. Women with such an obstetric history show higher rates of complications, including placenta previa, abruptio placentae, preterm premature rupture of membranes (PPROM), and admission to a neonatal intensive care unit (NICU) [4]. Even multiple first‑trimester abortions are linked to adverse outcomes, suggesting an underlying uterine or endocrine dysfunction [5].

Findings from Indian and international settings underscore how cultural, social, and healthcare access issues influence pregnancy outcomes after miscarriage. Research in rural and semi‑rural facilities shows that women who have had SABs tend to have greater anxiety and higher risks of hypertension during pregnancy and anemia [6]. In a recent Indian observational study, women with a previous SAB had a significantly increased risk of poor pregnancy outcomes, including gestational hypertension and preterm delivery [7]. Comparative hospital‑based studies confirm that women with one or more previous abortions have statistically significant differences in antenatal complications and mode of delivery compared with primigravidae [8]. Furthermore, the presence of antiphospholipid antibodies in women with early miscarriages has been strongly linked to an increased risk of thrombotic events and further miscarriages; if such immunological conditions are not diagnosed, they may contribute to a higher risk of recurrence and stillbirth [9].

The risks are not limited to the mother. Fetal well‑being is also compromised in subsequent pregnancies, mainly due to uteroplacental insufficiency, cervical incompetence, or an ongoing maternal inflammatory state. Women with a history of abortion are at increased risk of delivering a preterm or low‑birth‑weight infant or an infant requiring NICU admission, particularly after more than one loss [10]. Early abortions may be associated with elevated maternal stress levels that trigger neuroendocrine reactions, negatively affecting fetal development [11].

The COVID‑19 pandemic further highlighted the need for targeted maternal care for women with previous pregnancy losses, especially in the context of health inequities. Indirect effects of pandemic restrictions - reduced healthcare access, missed prenatal care, and psychosocial stressors - have added to the dangers in this population [12]. Despite these challenges, there is striking continuity across pregnancies: an SAB is a risk factor for adverse outcomes in the next pregnancy, justifying closer surveillance [13].

In summary, a history of SAB is not an isolated event but a potentially important risk factor for future obstetric outcomes. Women with such a history should be identified as a vulnerable group requiring differentiated care, enhanced pregnancy monitoring, and psychological support. However, despite advances in reproductive medicine, the long‑term impact of miscarriage on subsequent pregnancies remains an outstanding research question. This study aimed to observe the fetomaternal outcome of the present pregnancy following SAB.

## Materials and methods

Study design

This was a hospital‑based prospective observational study conducted in the Department of Obstetrics and Gynecology at a private tertiary care hospital, Puducherry. The study followed a prospective design, enrolling women attending the antenatal outpatient department (OPD) as well as those admitted to the labor ward with a history of SAB. Data were collected from the start of pregnancy and documented using a structured proforma designed according to the study protocol. Obstetric and neonatal outcomes were evaluated from the initial antenatal visit through the postnatal period.

Study setting

The study was carried out at a tertiary care hospital, Puducherry, in the Department of Obstetrics and Gynecology. Women were recruited from the OPD and the labor ward, covering all stages of pregnancy. All assessments, investigations, and follow‑up evaluations were performed using the clinical facilities available within the department. The setting provided access to a diverse obstetric patient population, routine antenatal care, emergency obstetric services, ultrasonography, laboratory services, and neonatal care facilities, enabling a comprehensive assessment of maternal and fetal health. The structured hospital environment facilitated systematic recruitment, examination, monitoring, and documentation according to the approved protocol.

Study duration

The study lasted for 18 months, as per the approved protocol. This period included the screening phase, enrollment phase, prospective follow‑up, intrapartum assessment, and postpartum evaluation. Women were enrolled on an ongoing basis throughout the data collection period until the desired sample size was achieved. The 18‑month timeline allowed sufficient observation of the natural course of pregnancy in women with and without a history of SAB, ensuring complete recording of all maternal and fetal outcomes. Follow‑up continued until delivery and the immediate postpartum period, capturing antenatal complications and neonatal parameters within the study window.

Participants

Inclusion criteria were women aged 18 to 35 years with at least one history of SAB (regardless of gestational age or cause) occurring before 20 completed weeks of gestation. Both first- and second-trimester SABs (<20 weeks) were included irrespective of the underlying cause. Exclusion criteria included previous induced abortion (regardless of whether it occurred before or after an SAB), uterine anomalies, the presence of any medical disorder, consanguineous marriage, previous twin gestations with a history of SAB, positive serology for HIV, hepatitis B surface antigen (HBsAg), or Venereal Disease Research Laboratory (VDRL) test, and refusal to provide informed consent. 

Sampling and sample size

The sample size was calculated based on a previous study [14], reporting 16.5% prevalence of adverse maternal outcomes. Considering 8% absolute precision, 95% confidence interval, and 80% power, the minimum required sample size was estimated to be 86. After adding a 10% non-response rate, the final sample size was rounded to 96 participants. A purposive sampling technique was used. Eligible antenatal women attending the OPD and labor ward were screened sequentially. Those meeting the inclusion criteria were recruited after obtaining written informed consent. 

Study parameters

Data collection focused on parameters reflecting maternal and fetal outcomes.

Primary Study Parameters

Maternal obstetric parameters: These included maternal age, gravida and parity, number of previous SAs, and the gestational age at which those losses occurred. Antenatal complications recorded were anemia, gestational hypertension, gestational diabetes mellitus (GDM), hyperemesis, and threatened abortion. Intrapartum parameters included onset of labor (spontaneous or induced), duration of labor, need for augmentation, and mode of delivery (normal vaginal delivery, instrumental delivery, or lower segment cesarean section). Maternal morbidity outcomes documented at delivery and during the immediate postpartum period included antepartum hemorrhage (placenta previa or abruption), intrapartum complications (fetal distress, meconium‑stained liquor), postpartum hemorrhage, hypertensive emergencies, and need for maternal ICU admission.

Secondary Study Parameters

Fetal and neonatal outcomes: These were recorded at birth, one hour after delivery, and at discharge. Variables included gestational age at delivery, birth weight (categorized as low birth weight <2.5 kg), Apgar scores at 1 and 5 minutes, and requirement for NICU admission. Early neonatal complications such as respiratory distress, hypoglycemia, and sepsis risk were also monitored. Additional comparative parameters specifically used to evaluate the impact of previous SAB on the current pregnancy included the incidence of preterm birth, the incidence of IUGR or small-for-gestational-age fetus, rates of cesarean section, rates of obstetric intervention, and the overall fetomaternal outcome score.

Study Procedure

After obtaining institutional ethical approval, women attending the antenatal OPD or admitted for delivery were screened consecutively against the inclusion and exclusion criteria. Once the obstetric history was confirmed, participants were categorized into the two study groups. Written informed consent was obtained from all eligible women before enrolment. Demographic information - including gravida, parity, socioeconomic factors, and booking status - was recorded using a structured proforma designed for the study. Detailed obstetric history, including the number, timing, and nature of previous pregnancy losses, was taken. Routine antenatal investigations (hemoglobin, blood group, Rh factor, urine examination, and obstetric ultrasound) were reviewed and recorded.

Participants were observed throughout pregnancy for complications such as anemia, gestational hypertension, GDM, and threatened abortion. Upon admission for labor, the following were recorded: cervical dilatation, onset of labor, indications for induction or augmentation, fetal heart rate characteristics, and liquor status, following departmental continuous monitoring protocols. Mode of delivery and indications for operative delivery were documented. Intrapartum complications, postpartum hemorrhage, hypertensive emergencies, and ICU admission were noted.

Immediately after birth, fetal outcomes were measured: gestational age, birth weight, Apgar scores at 1 and 5 minutes, need for neonatal resuscitation, and NICU admission. Early neonatal complications (respiratory distress, hypoglycemia, sepsis) were monitored until discharge. All parameters were coded for statistical analysis and entered on dedicated study sheets to ensure accuracy.

Ethical considerations

The study was approved by the Institutional Ethics Committee, Mahatma Gandhi Medical College and Research Institute (MGMCRI) (MGMCRI/Res/05/2023/80) before data collection commenced. All procedures complied with the principles of the Declaration of Helsinki and national guidelines for biomedical research on human subjects. Eligible women received a detailed explanation of the study, and written informed consent was obtained before enrolment. Participants were informed that participation was voluntary and that they could withdraw at any time without affecting the quality of care they received. 

Data collection

Data were collected systematically using the structured proforma. The following parameters were recorded: maternal demographic data (age, gravida, parity, socioeconomic and booking status); past pregnancy history (number of SABs and trimester of loss); antenatal parameters (hemoglobin, blood group, Rh factor, routine ANC investigations, ultrasound scans); pregnancy complications (anemia, gestational hypertension, gestational diabetes, threatened abortion); intrapartum information (onset of labor, mode of delivery, fetal heart rate status); maternal outcomes (intrapartum complications, postpartum hemorrhage, ICU care); and neonatal outcomes (birth weight, Apgar scores, need for resuscitation, NICU admission).

Data analysis

All collected data were checked for completeness and accuracy. Continuous variables (maternal age, gestational age, hemoglobin level, birth weight) were summarized as mean, standard deviation, and range. Categorical variables (parity, anemia, gestational hypertension, gestational diabetes, mode of delivery, neonatal outcomes) were presented as frequencies and percentages. The chi-square test was used to compare categorical variables between NICU and non-NICU admissions and between preterm and term deliveries. A *P*-value <0.05 was considered statistically significant.

## Results

A total of 96 pregnant women with a history of spontaneous abortion were included in the study. The mean maternal age was 27.74 ± 4.81 years, with ages ranging from 18 to 41 years. Most participants were aged <30 years (65/96, 67.7%), while 31 (32.3%) were aged ≥30 years. The majority of pregnancies resulted from spontaneous conception (88/96, 91.7%), whereas 6 (6.2%) pregnancies followed ovulation induction and 2 (2.1%) were conceived through in vitro fertilization (IVF). Regarding abortion history, 82 women (85.4%) had experienced a single previous spontaneous abortion, while 14 women (14.6%) had a history of recurrent spontaneous abortions (≥2). These findings indicate that the study population largely consisted of younger women with spontaneous conception and a single prior pregnancy loss (Table 1).

**Table 1:** Sociodemographic and obstetric characteristics of study participants (N = 96). IVF, in vitro fertilization

Characteristic	*n* (%)
Maternal age (years), mean ± SD	27.74 ± 4.81
Age ≥30 years	31 (32.3)
Age <30 years	65 (67.7)
Method of conception	
Spontaneous	88 (91.7)
Ovulation induction	6 (6.2)
IVF conception	2 (2.1)
Previous abortion history	
Single abortion	82 (85.4)
Recurrent abortion (≥2)	14 (14.6)

Among the maternal complications observed, GDM (17/96, 17.7%) was more frequent than gestational hypertension (12/96, 12.5%). Placental complications were uncommon, with placenta previa, abruptio placenta, and adherent placenta each occurring in 1 patient (1.0%). Membrane-related complications were infrequent, with both premature rupture of membranes (PROM) and preterm premature rupture of membranes (PPROM) reported in 4 women (4.2%) each. Regarding the mode of delivery, 58 women (60.4%) underwent lower segment cesarean section (LSCS), whereas 38 women (39.6%) had spontaneous vaginal delivery. No cases of malpresentation were documented. Additionally, 12 women (12.5%) delivered preterm. Overall, the maternal outcome profile was characterized by a relatively high cesarean section rate and a moderate prevalence of gestational diabetes and preterm birth (Table 2).

**Table 2:** Distribution of maternal pregnancy and delivery outcomes among the study participants (N = 96). PROM, premature rupture of membrane; PPROM, preterm premature rupture of membrane; LSCS, lower-segment cesarean section

Outcome	*n* (%)
Gestational hypertension	12 (12.5)
Gestational diabetes mellitus	17 (17.7)
Placenta previa	1 (1.0)
Abruptio placenta	1 (1.0)
Adherent placenta	1 (1.0)
PROM	4 (4.2)
PPROM	4 (4.2)
LSCS	58 (60.4)
Vaginal delivery	38 (39.6)
Malpresentation	0 (0.0)
Preterm delivery	12 (12.5)

Among fetal outcomes, fetal growth restriction (FGR) was observed in 12 neonates (12.5%), while no cases of intrauterine death (0%) were reported. The vast majority of newborns had favorable birth outcomes, with 95 neonates (99.0%) having a birth weight ≥2.5 kg and only 1 neonate (1.0%) classified as low birth weight. Similarly, 95 neonates (99.0%) had a normal Apgar score (7-10), whereas only 1 neonate (1.0%) had a low Apgar score (<7). Meconium aspiration syndrome was documented in 1 neonate (1.0%). A total of 18 neonates (18.8%) required NICU admission, making it the most common adverse neonatal outcome in the study. Despite the relatively high NICU admission rate, overall neonatal outcomes were favorable, as reflected by the low prevalence of low birth weight, low Apgar scores, and neonatal morbidity (Table 3).

**Table 3:** Distribution of fetal and neonatal outcomes among the study participants (N = 96). NICU, neonatal intensive care unit

Outcome	*n* (%)
Fetal growth restriction (FGR)	12 (12.5)
Intrauterine death (IUD)	0 (0.0)
Low birth weight (<2.5 kg)	1 (1.0)
Normal birth weight (≥2.5 kg)	95 (99.0)
Low Apgar score (<7)	1 (1.0)
Normal Apgar score (7–10)	95 (99.0)
Meconium aspiration syndrome	1 (1.0)
NICU admission	18 (18.8)

Among the 96 study participants, 12 (12.5%) experienced preterm delivery. The proportion of preterm birth was higher among women with recurrent spontaneous abortion (3, 21.4%) compared with those with a single previous abortion (9, 11.0%), although the association was not statistically significant (χ² = 0.79, *P* = 0.375). Preterm delivery occurred in 4 (12.9%) women aged ≥30 years and 8 (12.3%) women aged <30 years, with no significant association between maternal age and preterm birth (χ² = 0.01, *P* = 0.93). With respect to conception method, preterm birth was observed in 11 (12.5%) spontaneous conceptions and 1 (12.5%) assisted conception, showing no significant difference (1.87, *P* = 0.99). Women with GDM had a slightly higher frequency of preterm birth (3, 17.6%) compared with those without GDM (9, 11.4%), but the association was not statistically significant (*P* = 0.64). Similarly, preterm delivery occurred in 2 (16.7%) women with gestational hypertension and 10 (11.9%) women without gestational hypertension (*P* = 0.78). Preterm birth was recorded in 1 (25.0%) woman with PROM compared with 11 (12.0%) women without PROM (*P* = 0.58). Likewise, 2 (50.0%) women with PPROM experienced preterm delivery compared with 10 (10.9%) women without PPROM; however, this association was not statistically significant (*P* = 0.20). Overall, none of the evaluated maternal factors showed a statistically significant association with preterm delivery (all *P* > 0.05) (Table 4).

**Table 4:** Association of maternal characteristics with preterm delivery among the study participants (N = 96). Chi-square test/Fisher's exact test. *P*-value <0.05 is statistically significant. PROM, premature rupture of membrane; PPROM, preterm premature rupture of membrane

Variable	Category	Preterm, *n* (%)	Term, *n* (%)	χ² value	*P*-value*
Maternal age	<30 years (*n *= 65)	8 (12.3)	57 (87.7)	0.01	0.93
	≥30 years (*n *= 31)	4 (12.9)	27 (87.1)		
Previous abortion history	Single abortion (*n *= 82)	9 (11.0)	73 (89.0)	0.79	0.375
	Recurrent abortion ≥2 (*n *= 14)	3 (21.4)	11 (78.6)		
Method of conception	Spontaneous (*n *= 88)	11 (12.5)	77 (87.5)	1.87	0.99
	Assisted conception† (*n *= 8)	1 (12.5)	7 (87.5)		
Gestational diabetes mellitus	Absent (*n *= 79)	9 (11.4)	70 (88.6)	0.22	0.64
	Present (*n *= 17)	3 (17.6)	14 (82.4)		
Gestational hypertension	Absent (*n *= 84)	10 (11.9)	74 (88.1)	0.08	0.78
	Present (*n *= 12)	2 (16.7)	10 (83.3)		
PROM	Absent (*n *= 92)	11 (12.0)	81 (88.0)	0.30	0.58
	Present (*n *= 4)	1 (25.0)	3 (75.0)		
PPROM	Absent (*n *= 92)	10 (10.9)	82 (89.1)	1.64	0.20
	Present (*n *= 4)	2 (50.0)	2 (50.0)		

Among the 96 neonates, 18 (18.8%) required NICU admission, while 78 (81.2%) did not. NICU admission was more frequent among mothers with recurrent abortion history (4, 28.6%) compared with those with a single previous abortion (14, 17.1%), although the association was not statistically significant (χ² = 1.11, *P* = 0.291). Similarly, NICU admission occurred in 7 (22.6%) neonates born to mothers aged ≥30 years compared with 11 (16.9%) born to mothers aged <30 years (*P* = 0.57). A higher proportion of NICU admissions was observed among mothers with GDM (6, 35.3%) than among those without GDM (12, 15.2%). Although this association approached statistical significance, it did not reach the conventional threshold (χ² = 3.04, *P* = 0.08). Gestational hypertension was not associated with NICU admission, occurring in 3 (25.0%) affected pregnancies compared with 15 (17.9%) pregnancies without hypertension (*P* = 0.87). NICU admission was noted in 2 (25.0%) assisted conceptions and 16 (18.2%) spontaneous conceptions (*P* = 0.98). Similarly, higher NICU admission rates were observed among women with PROM (2, 50.0%) and PPROM (2, 50.0%) compared with those without these conditions; however, neither association was statistically significant (*P* = 0.40 and *P* = 0.09, respectively). Preterm delivery showed a significant association with NICU admission. NICU admission occurred in 5 (41.7%) preterm neonates compared with 13 (15.5%) term neonates (χ² = 4.08, *P* = 0.043). Thus, among the variables studied, preterm birth was the only factor significantly associated with NICU admission, while gestational diabetes demonstrated a trend toward increased NICU utilization (Table 5).

**Table 5:** Association of maternal characteristics with NICU admission among the study participants (N = 96). Chi-square test/Fisher's exact test. *P*-value <0.05 is statistically significant. PROM, premature rupture of membrane; PPROM, preterm premature rupture of membrane

Variable	Category	NICU admission, *n* (%)	No NICU admission, *n* (%)	χ² value	*P*-value*
Previous abortion history	Single abortion (*n *= 82)	14 (17.1)	68 (82.9)	1.11	0.291
	Recurrent abortion ≥2 (*n *= 14)	4 (28.6)	10 (71.4)		
Maternal age	<30 years (*n *= 65)	11 (16.9)	54 (83.1)	0.32	0.57
	≥30 years (*n *= 31)	7 (22.6)	24 (77.4)		
Gestational diabetes mellitus	Absent (*n *= 79)	12 (15.2)	67 (84.8)	3.04	0.08*
	Present (*n *= 17)	6 (35.3)	11 (64.7)		
Gestational hypertension	Absent (*n *= 84)	15 (17.9)	69 (82.1)	0.03	0.87
	Present (*n *= 12)	3 (25.0)	9 (75.0)		
Method of conception	Spontaneous (*n *= 88)	16 (18.2)	72 (81.8)	2.54	0.96
	Assisted conception† (*n *= 8)	2 (25.0)	6 (75.0)		
PROM	Absent (*n *= 92)	16 (17.4)	76 (82.6)	0.71	0.40
	Present (*n *= 4)	2 (50.0)	2 (50.0)		
PPROM	Absent (*n *= 92)	16 (17.4)	76 (82.6)	2.82	0.09*
	Present (*n *= 4)	2 (50.0)	2 (50.0)		
Preterm delivery	No (*n *= 84)	13 (15.5)	71 (84.5)	4.08	0.043*
	Yes (*n *= 12)	5 (41.7)	7 (58.3)		

## Discussion

This study examined the fetomaternal outcomes in women with a history of SAB who conceived a subsequent pregnancy. The findings demonstrate that while most women experienced favorable outcomes, including no intrauterine deaths, normal birth weight in the vast majority of neonates, and normal Apgar scores, clinically important morbidities such as GDM, gestational hypertension, fetal growth retardation, high cesarean section rates, and NICU admissions were observed. These results highlight that although a prior SAB should not cause undue alarm, it does warrant structured antenatal surveillance.

The mean maternal age in the study was 27.74 years, with a wide reproductive age range. Maternal age ≥30 years did not significantly influence GDM or preterm birth in this cohort, which contrasts with some studies that report advancing age as an independent risk factor for these complications. The lack of association may be due to the relatively small sample size or the fact that most women were in the active reproductive age group. Regarding the method of conception, the vast majority of women conceived spontaneously in the subsequent pregnancy, indicating that prior SAB does not generally impair future fertility. Assisted reproductive techniques were used in a small minority, and no significant association was found between conception type and placental complications. This is consistent with the reassuring message that natural conception remains the norm after a miscarriage.

The obstetric code distribution showed that most participants were either G2A1 or G3P1L1A1, reflecting a reproductive background of a single previous abortion with or without a live birth. Women with recurrent SAB (two or more losses) comprised about one‑sixth of the cohort. Recurrent abortion was associated with a higher rate of preterm delivery compared with a single abortion, although the difference did not reach statistical significance. Similar trends have been reported by Field and Murphy and Ausbeck et al., where recurrent miscarriage was linked to increased risks of preterm birth, intrauterine growth restriction, low birth weight, and NICU admission [15,16]. 

GDM was more prevalent than gestational hypertension in this study population, with rates of 17.7% and 12.5%, respectively. These figures are clinically significant and higher than those reported in some population‑based studies of women without previous pregnancy loss. For instance, Hautamäki et al. and Chen et al. found increased risks of GDM and hypertensive disorders in women with recurrent pregnancy loss [17,18]. The presence of GDM did not significantly affect mean birth weight in the present study, but it showed a trend towards increased NICU admission, suggesting that metabolic complications may impact neonatal well‑being through mechanisms other than altered birth weight, such as neonatal hypoglycemia or respiratory distress.

Placental complications were rare, with only single cases of placenta previa, abruptio placentae, and adherent placenta. This low incidence is reassuring and consistent with earlier reports by Gunnarsdottir et al. and Weintraub et al., who found only modest increases in placenta previa and abruption among women with prior miscarriage, mainly in those with three or more losses [19,20]. The absence of accreta spectrum cases further supports the conclusion that previous SAB alone is not a strong predictor of major placental morbidity. PROM and PPROM occurred in a small proportion of pregnancies (4.2% each). Membrane‑related complications were not significantly associated with abortion history, and the rates were lower than those reported in studies focusing on recurrent miscarriage, where higher incidences of preterm birth and antepartum hemorrhage have been documented [14-16]. This suggests that in a cohort predominantly composed of single SAB, membrane integrity is generally preserved.

The mode of delivery analysis revealed a high cesarean section rate (60.4%) despite the complete absence of malpresentation. This rate is substantially higher than reported in previous studies of pregnancy after miscarriage [21,22]. This may reflect the tertiary referral nature of our institution, where a greater proportion of high-risk pregnancies are managed, together with increased obstetric vigilance in women with a history of pregnancy loss. The discrepancy may reflect differences in clinical practice, increased obstetric caution due to previous pregnancy loss, maternal anxiety, or institutional protocols. Importantly, the high LSCS rate was not explained by fetal malpresentation, indicating that other maternal or fetal indications (e.g., previous cesarean, fetal distress, maternal medical conditions) likely drove the operative delivery decisions. The favorable neonatal outcomes, with only one low Apgar score and one case of meconium aspiration, suggest that the high operative rate did not translate into increased neonatal compromise. 

Fetal growth retardation was observed in 12.5% of pregnancies, a rate similar to that reported by Trilla et al. and higher than many other comparative studies [23-25]. This finding underscores the importance of serial fetal growth monitoring in women with a history of SAB, as placental insufficiency may be a shared pathway linking prior miscarriage to subsequent growth restriction. Encouragingly, intrauterine death did not occur in any case, and normal birth weight was achieved in 99% of neonates. The mean birth weight was identical in recurrent and single abortion groups (3,000 g), indicating that abortion history alone did not determine fetal size.

Neonatal outcomes were generally excellent, with 99% of neonates having normal Apgar scores and only one case of low birth weight. Meconium aspiration occurred in a single neonate. However, NICU admission was required in 18.8% of neonates, a rate higher than that reported in many previous studies [14-17]. The high NICU admission rate occurred despite predominantly normal birth weight and Apgar scores, suggesting that admissions were largely for observation, monitoring of neonatal hypoglycemia (in GDM pregnancies), prematurity, or growth concerns rather than severe birth depression. GDM showed a trend towards increased NICU admission risk, consistent with known neonatal effects of maternal diabetes. Preterm birth was more common in the recurrent abortion group (21.4%) than in the single abortion group (11.0%), although this difference was not statistically significant. This finding aligns with the literature, where recurrent miscarriage has been consistently associated with an increased risk of preterm delivery [15-19].

The study has several strengths, including a comprehensive assessment of both maternal and fetal outcomes, subgroup analysis by single versus recurrent SAB, and the use of inferential statistics to support clinical interpretation. However, limitations must be acknowledged. The small sample size, particularly the small number of women with recurrent abortion (*n* = 14), limited statistical power and may explain why some clinically important associations did not reach significance. The absence of a control group of women without any history of SAB precludes direct quantification of excess risk attributable to prior miscarriage. Additionally, several potential confounders (body mass index (BMI), socioeconomic status, thyroid disorders, thrombophilia, and detailed abortion characteristics) were not fully analyzed. The lack of BMI data prevented assessment of its relationship with Apgar scores and other outcomes. Furthermore, specific indications for cesarean section were not recorded, limiting the ability to understand the drivers of the high operative delivery rate.

Future research should include larger, multicenter prospective studies with adequate control groups to precisely quantify the risks associated with prior SAB. Detailed characterization of previous abortions (gestational age, trimester, need for surgical evacuation, infection history) and systematic collection of confounders (BMI, metabolic profile, autoimmune markers) would strengthen causal inference. The role of serial fetal growth monitoring, Doppler studies, and structured antenatal surveillance protocols should be evaluated in interventional studies to determine whether enhanced monitoring improves outcomes.

## Conclusions

In conclusion, this study demonstrates that while pregnancy after spontaneous abortion is generally associated with favorable outcomes, including survival and normal birth weight, there remain clinically significant risks of GDM, gestational hypertension, fetal growth retardation, high cesarean delivery, and NICU admission. Preterm, PROM, and GDM deliveries appear to amplify some of these risks, particularly NICU admissions. These findings support a balanced approach to antenatal care, reassurance regarding good outcomes combined with vigilant screening for metabolic and placental complications, thoughtful delivery planning, and preparedness for neonatal support.

## References

[1] Kashanian M, Akbarian AR, Baradaran H, Shabandoust SH (2006). Pregnancy outcome following a previous spontaneous abortion (miscarriage). Gynecol Obstet Invest.

[2] Patel K, Pirie D, Heazell AE, Morgan B, Woolner A (2024). Subsequent pregnancy outcomes after second trimester miscarriage or termination for medical/fetal reason: a systematic review and meta-analysis of observational studies. Acta Obstet Gynecol Scand.

[3] Sun H, Mao J, Su X, Du Q (2023). Impact of spontaneous abortion history and induced abortion history on perinatal outcomes of singleton pregnancies. BMC Public Health.

[4] Liu L, Zhang J, Wang R, Zhang W, Wang K, Wang F (2025). Relationship between prior pregnancy loss and subsequent adverse pregnancy outcomes in women. BMC Pregnancy Childbirth.

[5] Yang J, Wang Y, Wang XY, Zhao YY, Wang J, Zhao YY (2017). Adverse pregnancy outcomes of patients with history of first‐trimester recurrent spontaneous abortion. Biomed Res Int.

[6] Agrawal S, Agrawal V, Suhane R (2015). Pregnancy outcome following spontaneous abortions. Int J Reprod Contracept Obstet Gynecol.

[7] Rajurkar K (2023 ). Pregnancy outcome in women with a history of spontaneous abortion in previous pregnancy. Ind Obstet Gynaecol.

[8] Singh P, Gautam R, Jeyaseelan S (2023). A study on pregnancy outcome following previous spontaneous abortion: a hospital-based prospective observational comparative study. Int J Reprod Contracep Obstet Gynecol.

[9] Chauleur C, Galanaud JP, Alonso S (2010). Observational study of pregnant women with a previous spontaneous abortion before the 10th gestation week with and without antiphospholipid antibodies. J Thromb Haemost.

[10] Makhlouf MA, Clifton RG, Roberts JM (2014). Adverse pregnancy outcomes among women with prior spontaneous or induced abortions. Am J Perinatol.

[11] El-Raheem A, Khalaf AE, Mohamed AH (2022). Obstetric outcomes in women with threatened abortion. Al-Azhar Int Med J.

[12] Thom DH, Nelson LM, Vaughan TL (1992). Spontaneous abortion and subsequent adverse birth outcomes. Am J Obstet Gynecol.

[13] Yang X, Mu F, Zhang J, Yuan L, Zhang W, Yang Y, Wang F (2024). Reproductive factors and subsequent pregnancy outcomes in patients with prior pregnancy loss. BMC Pregnancy Childbirth.

[14] Kumari K, Misra M, Jhanwar A (2020). Fetomaternal outcome in twin pregnancies: a retrospective analysis from a tertiary care center. J Clin Diagn Res.

[15] Field K, Murphy DJ (2015). Perinatal outcomes in a subsequent pregnancy among women who have experienced recurrent miscarriage: a retrospective cohort study. Hum Reprod.

[16] Ausbeck EB, Blanchard C, Tita AT, Szychowski JM, Harper L (2021). Perinatal outcomes in women with a history of recurrent pregnancy loss. Am J Perinatol.

[17] Hautamäki H, Gissler M, Heikkinen-Eloranta J, Tiitinen A, Peuranpää P (2025). Pregnancy and perinatal outcomes in women with recurrent pregnancy loss-a case-control study. Acta Obstet Gynecol Scand.

[18] Chen Y, Chu B, Chen Y (2025). The effect of recurrent pregnancy loss on obstetric and neonatal outcomes: a systematic review and meta-analysis. J Matern Fetal Neonatal Med.

[19] Gunnarsdottir J, Stephansson O, Cnattingius S, Akerud H, Wikström AK (2014). Risk of placental dysfunction disorders after prior miscarriages: a population-based study. Am J Obstet Gynecol.

[20] Weintraub AY, Sergienko R, Harlev A, Holcberg G, Mazor M, Wiznitzer A, Sheiner E (2011). An initial miscarriage is associated with adverse pregnancy outcomes in the following pregnancy. Am J Obstet Gynecol.

[21] Ali N, Elbarazi I, Ghazal-Aswad S (2020). Impact of recurrent miscarriage on maternal outcomes in subsequent pregnancy: the Mutaba’ah study. Int J Womens Health.

[22] Muzaffar U, Rashid S, Salaam S (2020). Outcome of pregnancy following previous spontaneous abortion. Ind J Obstet Gynecol Res.

[23] Trilla C, Platero J, Camprubí N, Mora J, Luna C, Oros D, Llurba E (2025). First-trimester clinical characteristics and pregnancy outcomes in women with recurrent pregnancy loss. J Clin Med.

[24] Dadheech SK, Bharadwaj MK, Menon BA (2021). Pregnancy outcomes after spontaneous conception with previous spontaneous abortion preceding present pregnancy. Int J Reprod Contracep Obstet Gynecol.

[25] Panahi Z, Akbari R, Ghaemi M (2024). Recurrent pregnancy loss: a bibliometric review. Iran J Public Health.

